# Evaluation of an interactive education workshop on hospital pharmacists’ ethical reasoning: an observational study

**DOI:** 10.1186/s12910-024-01082-4

**Published:** 2024-07-23

**Authors:** Nallini McCleery, Adam La Caze, Karl Winckel, H. Laetitia Hattingh

**Affiliations:** 1 Pharmacy Department, Gold Coast Hospital and Health Service, 1 Hospital Blvd, Southport, QLD 4215 Australia; 2 School of Pharmacy, University of Queensland, Level 4, 20 Cornwall Street, Woolloongabba, QLD 4102 Australia; 3 Princess Alexandra Hospital, 199 Ipswich Rd, Woolloongabba, QLD 4102 Australia; 4 Allied Health Research, Gold Coast Health, Southport, QLD 4215 Australia; 5 School of Pharmacy and Medical Sciences, Griffith University, Southport, QLD 4215 Australia

**Keywords:** Hospital pharmacists, Ethical reasoning, Ethics education, Medication management, Privacy and confidentiality

## Abstract

**Background:**

Pharmacists are often faced with scenarios in practice that require application of ethical reasoning and decision-making skills. There is limited research on the ethical decision-making processes of hospital pharmacists. Pharmacists who are compassionate and put the interests of their patients first are thought to positively impact on patient care, but there are often complex health-care system pressures in the hospital setting that cause pharmacists to behave in ways that may conflict with professional values and behaviours. This multisite study aimed to evaluate an interactive education workshop on hospital pharmacists’ ethical reasoning skills and explore the need for ongoing training and support.

**Methods:**

This mixed-methods study was carried out across two health services including three hospitals. It incorporated a pre-workshop survey, a feedback survey immediately post-workshop and a third survey four weeks after the workshop. Semi-structured interviews were conducted with hospital pharmacists at least four weeks after the ethics workshop.

**Results:**

In total, 32 participants completed the pre-workshop survey, nominating peers/colleagues as the most common source of support they would consult to inform ethical decision-making (17/118 sources of support). Almost all (*n* = 31/33; 94%) *strongly agreed/agreed* that the education session provided them with ethical reasoning skills and a process/framework which they could use when faced with an ethical issue. Pre- and post-survey responses showed increased self-confidence in identifying the regulatory frameworks applicable to pharmacy privacy requirements (*p* = 0.011) and ethical issues applicable to pharmacy privacy requirements (*p* = 0.002), as well as applying ethical reasoning to scenarios that involve pharmacy privacy dilemmas/issues (*p* = 0.004). Participants’ self confidence in knowing where to find support when faced with clinical and non-clinical ethics questions was improved (*p* = 0.002 and *p* = 0.003 respectively). Participants supported the introduction of quarterly ethics cafes after the workshop, compared to before the workshop (*p* = 0.001).

**Conclusion:**

Hospital pharmacists rely on discussions with colleagues to brainstorm how to address ethical issues. This study showed that a targeted interactive education workshop facilitated familiarity with ethics resources and decision-making processes. It also demonstrated that this approach could be used to enhance hospital pharmacists’ readiness, confidence, and capabilities to recognise and respond to challenging ethical issues.

**Supplementary Information:**

The online version contains supplementary material available at 10.1186/s12910-024-01082-4.

## Background

Pharmacists are registered health professionals who are experts in medicines, practise independently and accept professional responsibility for patient care [1]. They are faced with dilemmas in practice that require application of ethical reasoning and decision-making skills. The Code of Ethics set out by the International Pharmaceutical Federation recognises the need for pharmacists to apply sound ethical principles to guide decisions in day-to-day practice [2]. The Pharmacy Board of Australia (PBA) defines pharmacists’ professional obligations through the Code of Conduct for Pharmacists that outlines acceptable professional behaviour and ethical standards for the profession [1]. The Pharmaceutical Society of Australia (PSA) developed the PSA Code of Ethics to guide pharmacists in varied settings, while the Society of Hospital Pharmacists Australia (SHPA) developed the SHPA Code of Ethics to specifically guide hospital pharmacy practice around ethical considerations [3, 4]. Professional Codes of Ethics (such as the PBA, PSA, and SHPA codes) identify key principles of pharmacy practice, but tend to be limited in relation to specific guidance for pharmacists facing the wide range of ethical challenges that present in practice.

Hospital pharmacy positions in Australia are increasing: between 2014 and 2017 there was a 22% increase in the number of pharmacists transferring from community to hospital practice [5]. Although both hospital and community pharmacy practices focus on patient-centred care as the core of pharmacy services provided, there are some differences in the settings and overall scope: hospital pharmacists mostly work in the acute setting with vulnerable populations and high-risk medicines, while community pharmacists focus on the management of patients in primary care, many of whom have chronic disease and complex needs [6]. Ethical challenges present in different ways in different professional contexts for hospital and community pharmacists. Community pharmacists may be more likely to experience ethical dilemmas regarding medicine dispensing, the supply of over-the-counter and complementary medicines and business administration, whereas hospital pharmacists may be more likely to experience ethical challenges relevant to complex medication management options whilst working within multidisciplinary teams [7–11]. For community pharmacists, the main dilemmas commonly arose when their professional autonomy was challenged by the behaviour of patients and other health professionals [12]. In a 2021 Australian study, hospital pharmacists outlined the challenges when moving from community to hospital practice [7]. In addition to general pharmacy ethical reasoning skills, hospital pharmacists therefore require targeted ethical training that is unique to their hospital setting where patients are often more unwell, could be acutely delirious or may lack capacity to understand or consent to decisions around medicines, therefore requiring complex medication management.

Recent studies undertaken in Australia have explored pharmacists’ ethical reasoning and decision-making processes [7, 8, 13]. Although hospital pharmacists encounter ethical decision-making as part of everyday hospital practice, it has been referred to as an ‘ethical grey zone’ when compared to the more frequently discussed challenges that arise in community pharmacy practice [7]. Pharmacists who are not confident in ethical decision-making could experience moral distress, which occurs when a practitioner’s own moral considerations conflict with institutional constraints [14, 15]. Pharmacy ethics research predominantly focuses on ethical difficulties faced by community pharmacists including the frequency and severity of moral distress [16, 17]. There is limited evidence on how hospital pharmacists make ethical decisions in contrast to other hospital-based practitioners such as nursing and medicine [16]. In addition, there is a lack of research that explores the value of targeted hospital pharmacy ethical training. A 2018 study conducted in Western Australia explored pharmacists’ and pharmacy students’ ethical reasoning skills [8]. The authors suggested that a structured ethical decision-making process could be of benefit, considering the diverse and ever-changing landscape of pharmacy practice, together with variations in ethical case complexity.

Research that incorporates ethical training interventions in pharmacy mainly focuses on pharmacy students [16]. Although this training is important to students’ professional development in gaining ethical reasoning skills, students may have limited hospital pharmacy experience to contextualise and apply the theoretical ethical training. Alongside these concerns is the lag in experience between university training and clinical practice. Ethical dilemmas relevant during undergraduate university training may no longer be relevant years later. One example of this, in Australia, is moving public opinions, expectations and changed legislation around voluntary assisted dying that has occurred in a very short period of time [18].

Moral case deliberation has been developed and studied within the clinical ethics support literature [19, 20]. Moral case deliberation involves health professionals meeting to collaboratively discuss a concrete case that raises questions that require ethical deliberation. These sessions are facilitated by an individual able to encourage participants to discuss and work through the challenges that arise. Moral case deliberation has been shown to support health professionals to discuss ethical issues, improve participants’ understanding of the perspectives of colleagues, and feel more confident in relation to responding to ethical challenges [19].

Research into ethics in pharmacy practice in the United Kingdom suggested pharmacists would benefit from ongoing formal ethical education to provide them with the necessary skills to assess an ethical dilemma, evaluate possible responses and justify the response adopted [21]. A 2006 Swedish study that explored moral distress in pharmacy suggested that training and education of staff should include content around ethical theory and incorporate discussion of morally stressful situations to develop staff’s ethical decision-making skills and management of morally stressful situations [22]. Three-quarters of pharmacists surveyed in a 2016 Croatian study felt that they were not adequately trained to make ethical decisions [23]. A 2021 Australian study identified a gap in education received by hospital pharmacists on ethical decision-making processes, and suggested that hands-on, practical, group learning sessions using case-based scenarios to provide a structured approach may be a solution [7]. There is evidence that interactive learning sessions stimulate higher level learning skills and the ability to incorporate skills into practise [24].

A need was identified to develop and evaluate an ethics training workshop targeted at hospital pharmacy practice through interactive learning sessions. Recent changes to legislation allowing voluntary assisted dying and specifying the pharmacists’ role within this, as well as changes to regulations regarding controlled drugs (medicines with abuse potential) emphasized the need for ongoing discussion and training in professional ethics [25, 26]. A workshop was designed to develop confidence and skills in dealing with ethical dilemmas for hospital pharmacists and hospital pharmacy interns in Australia. We aimed to evaluate the interactive education workshop on hospital pharmacists’ ethical reasoning skills and explore the need for ongoing training and support.

## Methods

This mixed-methods study incorporated surveys completed anonymously and semi-structured interviews with hospital pharmacists before and after an ethics workshop. The STrengthening the Reporting of OBservational studies in Epidemiology (STROBE) checklist and Consolidated Criteria for Reporting Qualitative Studies (COREQ) criteria was used to develop and report the findings of the surveys and interviews [27, 28].

### Ethics approval

Ethics approval was granted by the Gold Coast Hospital and Health Service (GCHHS) Human Research Ethics Committee (LNR/2021/QGC/81834) with reciprocal approval by Metro South Hospital and Health Service (MSHHS).

### Research setting

This study was undertaken across two Queensland Hospital and Health Services: GCHHS (Gold Coast University and Robina Hospitals) and MSHHS (Princess Alexandra Hospital (PAH)). Interactive education workshops were conducted at the Pharmacy Departments of the three hospitals.

### Study design

**Fig. 1:**
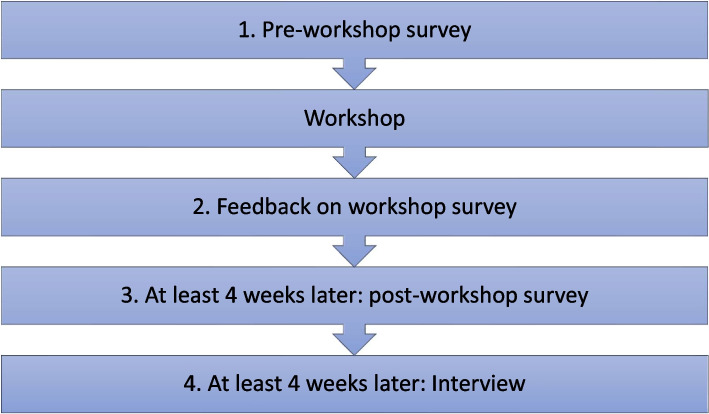
Overview of the study design and evaluation

Figure 1 provides an overview of the study. Workshop participants were requested to complete: 1) a pre-workshop survey, 2) a survey to obtain feedback on the workshop at the end of the session, and 3) a post-workshop survey four weeks later; and additionally, 4) workshop participants that indicated interest in participating in post workshop interviews were approached for semi-structured interviews at least four weeks after the workshop.

### Participants

Hospital pharmacists and pharmacy interns who worked at the three hospitals were offered the opportunity to attend an interactive education workshop. Workshop attendees were invited to participate in the study and provided with a participant information sheet; time was allocated to complete the pre-workshop survey at the start and feedback on workshop survey at the conclusion of the sessions. Participants were then emailed and invited to complete the post-workshop survey at least four weeks after workshops. Workshop participants were invited to interviews at least four weeks after workshops. Participants reviewed and completed participant information consent forms prior to being interviewed and responses recorded.

### Intervention

The intervention consisted of a 1-h workshop that focused on ethical reasoning. The discussion was prompted by a case, which highlighted medicines management, the challenge of respecting patient autonomy, working within a multidisciplinary team, and appropriately interpreting privacy legislation and hospital policy within a relatively complex clinical context (Table 1).

**Table 1:** Overview of the case and questions used to facilitate discussion during the interactive education workshop

In completing a pharmacist medication history with a patient, they disclose that they’re taking valaciclovir for genital herpes prophylaxis. The patient reveals that you are the only health professional they have mentioned this to whilst in hospital. It has not been charted. The patient has brought in their own medicine supply to take as normal with the nursing staff’s knowledge. The patient is adamant that you do not document this medicine in her records as her partner was unaware of the diagnosis. The patient’s liver function tests indicate derangement, and the treating team are unsure of the cause.
* 1. Identify the problem and possible consequences of the problem.*
* 2. Identify relevant law, codes and professional standards that apply to the case.*
* 3. Identify the available options for resolving the problem and the reasons for or against each one.*
* 4. Formulate a plan of action to resolve the problem.*

The workshop provided a framework for ethical decision-making adapted from Winch et al. 2014 (see Additional file 1 with more detail) [29]. The framework prompted participants to consider the ethical challenges presented within the case; the legal and professional guidance available to support decisions; the pros and cons of various professional responses to the case; and the development of an action plan. The workshop design and delivery was informed by a pedagogical schema for critical thinking, this included planning in the language of student cognition, shifting the focus from knowledge to inquiry and working collaboratively when thinking can be shared [30].

The following learning objectives guided the workshop content:

- Identify the regulatory framework and ethical principles applicable to a pharmacy privacy scenario
- Apply the regulatory requirements and ethical principles to the scenario to determine appropriate actions
- Consider the positive impact of using structured ethical decision-making skills on patient outcomes and interactions with other health professionals
- Develop and apply a structured ethical decision-making process to ethical dilemmas
- Discuss ethical challenges with peers

The discussion focussed on getting participants to share their experiences and perspectives in relation to the case. The facilitator sought to encourage individual and group reasoning in relation to the case. This included identifying assumptions and encouraging group discussion to clarify and evaluate reasons for adopting particular approaches within the case.

Various frameworks were integrated into the development of the training content. This included the PSA competency standards and the regulatory framework that highlights the order of documents to consult when confronted with ethical dilemmas [31], The PBA Code of Conduct [1], PSA Code of Ethics [4] and the 2021 Medicines and Poisons Regulation (Queensland) [26], as well as other relevant regulatory documents such as Privacy Principles [32]. Activities within the workshop were designed to support and encourage knowledge application rather than merely knowledge acquisition [24].

### Data collection

#### Surveys

Three surveys were developed: 1) a pre-workshop survey to obtain baseline data on participants’ ethical reasoning processes, 2) a survey immediately post-workshop to obtain feedback and 3) a 4-week post-workshop survey. Survey questions were developed using the previous Australian study survey as a basis [13], considering feedback from the 2021 hospital pharmacists’ study [7], as well as adding questions to address the changes in the legislation [25, 26]. Responses from the pre- and post-workshop surveys were compared for self-confidence ratings and responses to scenarios. Validation of surveys was achieved by inviting four pharmacists who were not involved in the study to review and comment on the surveys with feedback incorporated.

1. The pre-workshop survey consisted of five sections to capture participant demographic data, prioritisation of resources used to solve ethical scenarios, self-rated confidence with ethical reasoning and responses to practice scenarios that included privacy and confidentiality. There were 12 questions in total consisting of five Likert-style questions and seven open response questions [33]. Two Likert-scale questions determined frequency (*daily-monthly-never*) and three questions determined agreement with statements (*strongly disagree-agree-not sure*). An additional file shows this in more detail [see Additional file 2].
2. The survey to determine feedback on the workshop comprised three questions, one Likert-style (*strongly disagree-neither disagree nor agree-not relevant to role*), one open response and one multiple-choice.
3. The 4-week post-survey was similar to the pre-workshop survey, consisting of two repeated sections; participant self-rated confidence with ethical reasoning and responses to practice scenarios to determine changes to responses since delivery of the workshop.

Pre-workshop and feedback surveys were developed and delivered using hard copy. The 4-week post workshop surveys were emailed to participants who completed the survey in hard copy.

#### Interviews

Semi-structured interviews consisted of open-ended questions designed to explore perspectives on the workshop and its potential impact on practice, as well as responses when presented with specific ethical scenarios. The initial four questions captured participant demographic data. The following seven questions offered the opportunity to gain a broad understanding of participant exposure to ethical scenarios. These were followed by two practice scenarios to gain insights into how participants would approach an ethical challenge in practice. An additional file shows this in more detail [see Additional file 3].

### Data analysis

Survey results were extracted into Microsoft Excel and analysed descriptively and pre-post data using paired t-tests. Likert scales were translated into numerical values. The data was imported into Stata for descriptive analysis, including means and standard deviations for the continuous variables. A t-test was conducted to compare participant’s pre- and post-questionnaire scores. A *p*-value of < 0.05 was considered to represent statistical significance. Interview data was analysed descriptively. Responses were transcribed, deidentified and coded.

## Results

Five workshops were facilitated by research team members between March-June 2022. Three workshops were conducted face-to-face and two were provided in a hybrid format (face-to-face and Microsoft Teams). A total of 39 staff attended the workshops.

### Pre-and post-surveys

Thirty-three participants completed the pre-workshop surveys (84.6%). There were 26 hospital pharmacists and five pharmacy interns. One pharmacy student attended during the student’s university placement and one attendee did not identify their role (Table 2).

**Table 2:** Summary of participants who completed the pre-workshop survey

**Demographic**	**Number (*n* = 33)**	**%**
Gender		
Male	6	18.2%
Female	25	75.8%
Other	0	0%
Prefer not to say	0	0%
No response	2	6.1%
Age Group (years old)		
21-30	16	48.5%
31-40	12	36.4%
41-50	1	3.0%
51-60	3	9.1%
> 61	0	0%
No response	1	3.0%
Health Service Employment		
GCHHS^a^	20	60.6%
MSHHS^b^	13	39.4%
Position within Health Service		
Pharmacist	26	78.8%
Pharmacy Intern	5	15.2%
Pharmacy Student	1	3.0%
No response	1	3.0%
Years of experience as a pharmacist		
0 (intern/student)	6	18.2%
0-2	3	9.1%
> 2-5	7	21.2%
> 5-10	9	27.3%
> 10	7	21.2%
No response	1	3.0%
Years worked as a pharmacist at current Health Service		
0 (intern/student)	6	18.2%
0-2	6	18.2%
> 2-5	8	24.2%
> 5-10	8	24.2%
> 10	3	9.1%
No response	2	6.1%
Current Health Practitioner (HP) Level		
Pharmacy Student	1	3.0%
Pharmacy Intern	5	15.2%
HP3	13	39.4%
HP4	8	24.2%
HP5	4	12.1%
> HP6	1	3.0%
No response	1	3.0%

^a^*GCHHS* Gold Coast Hospital and Health Service

^b^*MSHHS *Metro South Hospital and Health Service

### Resources used

Participants were asked to nominate five sources of support they were likely to consult to inform their ethics decision-making. In total, participants listed 118 potential sources of support. The most common source of support referred to was peers/colleagues (17/118) followed by professional protocols or guidelines (11/118). This was followed by legislation, Codes, Director of Pharmacy/Deputy Director of Pharmacy/Manager and team leader/members, all (9/118).

When asked how often participants used their knowledge around the following resources to inform ethical decision making, co-workers within the pharmacy department were most commonly referred to as a resource on a daily basis (*n* = 13/32; 40%) and professional protocols and guidelines were also used on a daily basis, albeit by a smaller number (*n* = 7/32; 21%) (Table 3). Other resources such as professional indemnity insurers were used commonly but less frequently (Table 3).

**Table 3:** Resources used to inform ethical decision making (either from memory or looking up the resource)

**Resources**	**Daily**		**Weekly**		**Monthly**		** < Monthly**		**Never**	
	**n**	**%**	**n**	**%**	**n**	**%**	**n**	**%**	**n**	**%**
Codes of Ethics and Conduct	2	6.1%	2	6.1%	4	12.1%	15	45.5%	10	30.3%
Standard for the Uniform Scheduling of Medicines Poisons and relevant Commonwealth, state or territory drugs and poisons legislation	6	18.2%	0	0%	9	27.3%	13	39.4%	5	15.2%
Privacy Act (Cth) 1988 and privacy resources	3	9.1%	4	12.1%	3	9.1%	13	39.4%	10	30.3%
Legislation e.g. Medicines and Poisons Act (Qld) 2019 and Regulations 2021	5	15.2%	1	3.0%	14	42.4%	9	27.3%	3	9.1%
Professional Competency Standards	4	12.1%	0	0%	8	24.2%	15	45.5%	6	18.2%
Professional Practice Standards	4	12.1%	0	0%	8	24.2%	14	42.4%	7	21.2%
Professional protocols or guidelines	7	21.2%	4	12.1%	9	27.3%	8	24.2%	5	15.2%
Pharmacy Board of Australia Standards, Codes, Guidelines	1	3.0%	4	12.1%	7	21.2%	14	42.4%	7	21.2%
Workplace legal officer/team	1	3.0%	2	6.1%	2	6.1%	5	15.2%	23	69.7%
Workplace ethicist	1	3.0%	1	3.0%	1	3.0%	1	3.0%	29	87.9%
Co-workers in pharmacy department	13	39.4%	12	36.4%	3	9.1%	4	12.1%	0	0.00%
Co-workers in hospital outside of pharmacy department	4	12.1%	10	30.3%	5	15.2%	7	21.2%	7	21.2%
Discussion with colleagues via profession specific social media	0	0%	4	12.1%	2	6.0%	7	21.2%	20	60.6%
Staff at professional organisations (e.g. SHPA, PSA, Guild)	0	0%	0	0%	3	9.1%	11	33.3%	19	57.6%
Staff at the Pharmacy Board of Australia or AHPRA	0	0%	0	0%	1	3.0%	9	27.3%	23	69.7%
Professional indemnity insurer	0	0%	0	0%	1	3.0%	13	39.4%	19	57.6%

*SHPA* Society of Hospital Pharmacists of Australia, *PSA* Pharmaceutical Society of Australia, *AHPRA* The Australian Health Practitioner Regulation Agency

### Exposure to privacy and confidentiality scenarios

Participants were asked to indicate the frequency of exposure to ethical issues involving privacy and confidentiality scenarios in practice (Table 4). The most frequently encountered daily scenario was “a staff member on an in-patient unit leaves the computer screen on with identifiable patient information visible” (*n* = 11/33; 33%)) with the next most encountered scenario being “a patient is counselled on their medicines in front of a family member without obtaining their consent first” at (*n* = 6/33; 18%). Most participants (*n* = 23/33; 70%) were exposed to a scenario in which a patient requested that either they or another staff member withhold recording some of their medicine in the electronic health record.

**Table 4:** Frequency of exposure to privacy and confidentially scenarios in regular practice

**Scenarios**	**Daily**		**Weekly**		**Fortnightly**		**Monthly**		** < Monthly**		**Never**	
	**n**	**%**	**n**	**%**	**n**	**%**	**n**	**%**	**n**	**%**	**n**	**%**
The pharmacy receives a fax from a health professional intended for another receiver. The fax contains patient identifiers/information	4	12.1%	5	15.2%	3	9.1%	4	12.1%	11	33.3%	6	18.2%
A staff member relays medicines information to a family member and then realises that he/she may not be entitled to that information	2	6.1%	3	9.1%	4	12.1%	6	18.2%	13	39.4%	5	15.2%
A staff member shows one member of a family another family member's discharge medication record without obtaining consent from them	4	12.1%	7	21.2%	6	18.2%	5	15.2%	7	21.2%	4	12.1%
A patient is counselled on their medicines in front of a family member without obtaining their consent first	6	18.2%	11	33.3%	3	9.1%	4	12.1%	7	21.2%	2	6.1%
A patient requests you or another staff member to withhold recording some of their medicine in ieMR	0	0%	0	0%	2	6.1%	3	9.1%	18	54.6%	10	30.3%
A staff member discusses confidential de-identified information about a consumer outside of the pharmacy at a non-professional setting	0	0%	4	12.1%	2	6.1%	6	18.2%	14	42.4%	7	21.2%
A staff member discloses confidential identifiable information about a consumer(s) outside of the pharmacy	0	0%	2	6.1%	1	3.0%	7	21.2%	9	27.3%	14	42.4%
A staff member discloses real practice scenarios on a social media platform such as Facebook	2	6.1%	1	3.0%	1	3.0%	0	0%	6	18.2%	23	69.7%
A staff member sorts through prescriptions on a front/dispensary counter view of other consumers	1	3.0%	5	15.2%	2	6.1%	2	6.1%	7	21.2%	16	48.5%
Identifiable patient and/or consumer information disposed of in unsecured rubbish (e.g. note)	1	3.0%	4	12.1%	2	6.1%	3	9.1%	12	36.4%	11	33.3%
Empty, used dose administration aid (DAA) packs with identifiable header cards and medicine details are disposed of in unsecured rubbish	1	3.0%	1	3.0%	2	6.1%	4	12.1%	12	36.4%	13	39.4%
Medicines awaiting collection have dispensing labels visible to other consumers	2	6.1%	1	3.0%	1	3.0%	2	6.1%	15	45.5%	12	36.4%
A staff member on an in-patient unit leaves the computer screen on with identifiable patient information visible	11	33.3%	10	30.3%	5	15.2%	2	6.1%	3	9.1%	2	6.1%
Medicines handed out are visible to other patients in the in-patient unit	4	12.1%	2	6.1%	5	15.2%	9	27.3%	8	24.2%	4	12.1%

### Workshop feedback

Participants were asked to provide feedback immediately after workshops (Table 5). Almost all (*n* = 32/33; 97%) *strongly agreed/ agreed* that the scenario discussed in the workshop was relevant to their practice and the education session assisted in identifying ethical reasoning resources. Almost all (*n* = 31/33; 94%) *strongly agreed/agreed* that the education session provided them with ethical reasoning skills, a process/framework which they could use when faced with an ethical issue and the session was useful.

**Table 5:** Feedback provided immediately post-workshop

**Statement**	**Strongly disagree/disagree**		**Neither disagree nor agree**		**Agree/strongly agree**		**Not relevant to role**	
	**n**	**%**	**n**	**%**	**n**	**%**	**n**	**%**
The case scenario was relevant to my practice	1	3.0%	0	-	32	97.0%	0	-
The education session provided me with ethical reasoning skills	1	3.0%	0	-	31	93.9%	1	3.0%
The education session assisted me in identifying ethical reasoning resources	1	3.0%	0	-	32	97.0%	0	-
The education session provided me with a process/framework which I will be able to use when I am faced with an ethical issue	1	3.0%	1	3.0%	31	93.9%	0	-
The session has been useful	1	3.6%	1	3.6%	26	93.9%	0	-

### Confidence in ethical decision-making

Participants were asked twelve Likert rating type questions in both the pre-survey as well as 4-week post-workshop survey to identify self-confidence with a series of statements around ethical reasoning (Table 6). There were improvements between pre- and post-survey responses in self-confidence in identifying the regulatory frameworks applicable to pharmacy privacy requirements (*p* = 0.011), identifying the ethical issues applicable to pharmacy privacy requirements (*p* = 0.002), in applying ethical reasoning to scenarios that involve pharmacy privacy dilemmas/issues (*p* = 0.004). Also, participants’ self confidence in knowing where to find support when faced with clinical and non-clinical ethics questions was improved (*p* = 0.002 and *p* = 0.003 respectively). Participants identified they would be more likely to participate in quarterly ethics cafes after the workshop compared to before the workshop (*p* = 0.001).

**Table 6:** Self confidence in ethical decision-making skills

**Please rate your level of agreement with the following statements**	**Mean pre** **(95% CI)**	**Mean post** **(95% CI)**	***p*-value**
I am confident in identifying the regulatory frameworks applicable to pharmacy privacy requirements	3.13 (2.79-3.46)	3.87 (3.37-4.36)	0.011*
I am confident in identifying the ethical issues applicable to pharmacy privacy requirements	3.43 (3.13-3.73)	4.10 (3.69-4.15)	0.002*
I am confident in applying ethical reasoning to scenarios that involve pharmacy privacy dilemmas/issues	3.64 (3.39-3.88)	4.18 (3.83-4.52)	0.004*
I follow a structured ethical decision-making process when confronted with ethical dilemmas/issues	3.20 (2.86-3.54)	3.64 (3.19-4.10)	0.056
I know where to find support when I have a clinical ethics question	3.40 (3.08-3.72)	4.16 (3.71-4.61)	0.002*
I know where to find support when I have a non- clinical ethics question	3.41 (3.08-3.74)	4.12 (3.66-4.58)	0.003*
I discuss ethically challenging scenarios in my hospital practice with my peers	3.80 (3.40-4.21)	3.59 (2.99-4.19)	0.574
Structured ethical decision-making skills impact positively on patient outcomes	4.37 (4.07- 4.66)	4.67 (4.22-5.11)	0.269
Structured ethical decision-making skills impact positively on interactions with other health professionals	4.39 (4.16-4.63)	4.66 (4.31-5.02)	0.206
I am regularly presented with clinical ethics scenarios in my practice as a hospital pharmacist or intern	3.67 (3.36-3.98)	3.73 (3.27-4.19)	0.829
I am regularly presented with non-clinical ethics scenarios in my practice as a hospital pharmacist or intern	3.51 (3.18-3.83)	3.74 (3.27-4.21)	0.363
I would participate in quarterly ethics cafes (interactive small group discussions) if these were made available	3.97 (3.74-4.20)	4.67 (4.32-5.01)	0.001*

^*^A *p*-value of < 0.05 considered statistically significant

Statements that failed to show improvements were “I am regularly presented with clinical ethics scenarios in my practice as a hospital pharmacist or intern.”, which was unlikely to change as a result of the workshop and “I discuss ethically challenging scenarios in my hospital practice with my peers.”, which potentially may have been reduced as a result of attendees feeling more confident to manage ethical scenarios following the workshop.

### Management of ethical scenarios

Participants were asked in both pre- and post-surveys to review two ethical scenarios and rate their level of agreement with a series of statements using a Likert scale rating (Table 7). The first scenario centered on a palliative patient and a high dose of opioid. There was a significant change following the workshop where more participants agreed that they would discuss concerns with the patient and her husband (*p* = 0.028). There were no significant differences in responses of the second scenario (disagreeing with doctor’s decision) although there was an improvement in documenting decisions (*p* = 0.076).

**Table 7:** Management of ethical scenarios

**Palliative care patient and high dose of opioid** Scenario of hospital pharmacist reviewing a patient’s medicine prescribed for end-stage metastatic cancer. The patients’ husband does not want her to take any sedating pain medicines, even though the patient suffers from severe abdominal pain. The doctors prescribed regular opioids for the pain but the nurses have to withhold it when her husband is there. However, when he is not there at night, she requests the opioid and appears to be much more comfortable. When you talk to the patient during your inpatient unit review one morning, (the husband is not there, he has left to get a coffee), the patient tells you that her pain is intolerable, but she wants to ‘please’ her husband. You have another discussion with the nurse who tells you she has been ‘sneaking in’ when the husband is not there to administer some medicines as the patient is competent to make decisions.			
**Please indicate your level of agreement with the following hypothetical options you could undertake** You:	**Mean pre (95% CI)**	**Mean post** **(95% CI)**	***p*-value**
… agree with the nurse and would suggest she keeps doing the same thing with no need to inform the husband	2.74 (2.42-3.06)	2.86 (2.40-3.31)	0.628
… agree with the nurse and would suggest she keeps doing the same thing but insist the nurse informs the husband about this	2.76 (2.41-3.12)	2.73 (2.28-3.18)	0.885
… disagree with the nurse’s behaviour but do not interfere with the process as it is not your role	2.17 (1.96-2.38)	2.07 (1.77-3.27)	0.563
… inform the husband of the situation as he has legal say as the next of kin, do not report the incident	1.85 (1.60-2.10)	1.66 (1.30-2.03)	0.392
… inform the husband of the situation as he has legal say as the next of kin, report the incident	2.20 (1.81-2.58)	2.31 (1.74-2.88)	0.739
… do not discuss the situation with the patient or husband and Riskman or report an incident	1.74 (1.48-2.01)	2.20 (1.82-2.56)	0.024
…discuss your concerns with the patient but not the husband	3.69 (3.31-4.06)	4.02 (3.50-4.53)	0.228
… discuss your concerns with the patient and her husband	3.13 (2.75-3.51)	3.85 (3.29-4.41)	0.028*
… discuss the situation with a senior medical officer	4.43 (4.14-4.71)	4.52 (4.11-4.93)	0.698
… discuss the situation with another pharmacist	4.36 (4.05-4.67)	4.37 (3.91-4.82)	0.986
**Disagreeing with doctor’s decision** A patient has been given a discharge prescription for an antibiotic. The patient has not started the antibiotic in hospital. When you check his notes, you read that he previously had nausea and vomiting from an antibiotic in the same class resulting in non-compliance. You contact the discharge doctor, and she informs you that she was aware of the elderly gentleman’s previous experience, but she considered other possibilities and is content with her choice of medication. The doctor informs you she has met the patient on previous admissions, and in her opinion his adverse reaction is “not real, it’s all in his mind”.			
**Please rate your level of agreement with the following statements: after reading the case above:** You continue with discharge preparation:	**Mean pre** **(95% CI)**	**Mean post** **(95% CI)**	***p*-value**
… without saying anything to the patient because you do not want to discredit the doctor	1.73 (1.47-1.99)	1.68 (1.29-2.06)	0.837
… without saying anything to the patient because you accept the doctor’s explanation of the adverse reaction being all in the patient’s mind	1.70 (1.43-1.96)	1.62 (1.23-2.00)	0.720
… without saying anything to the patient as his previous adverse reaction was not serious	1.66 (1.47-1.84)	1.70 (1.45-1.96)	0.746
… without saying anything to the patient as it is more important for the patient to be compliant with his medication and telling him of the side effects may result in him being non-compliant	1.75 (1.49-2.01)	1.86 (1.49-2.24)	0.584
… and inform the patient that both medications are similar and therefore he may experience the same response as previously	4.03 (3.81-4.26)	4.00 (3.66-4.33)	0.878
… and you inform the patient that the medications are not similar and therefore it is unlikely for him to experience the same response as previously	1.49 (1.24-1.73)	1.73 (1.37-2.09)	0.246
… but discuss the situation with another pharmacist	3.54 (3.17-3.91)	4.00 (3.45-4.54)	0.188
… but discuss the situation with another medical officer	3.25 (2.86-3.63)	3.51 (2.94-4.08)	0.446
… but document your concerns and actions	3.57 (3.17-3.97)	4.15 (3.57-4.72)	0.076

^*^A *p*-value of < 0.05 considered statistically significant

### Interviews

Interviews were conducted with nine participants and provided an opportunity to confirm and/or further explore the quantitative survey responses. Two participants were intern pharmacists, three were HP3 pharmacists and four were HP4 pharmacists. The discussion topics of the interviews aligned well with the findings from the survey.

All agreed that they had developed their ethical reasoning skills through practice. This is evidenced by statements such as (P7): “In my work place, we have a little tea room and we often sit around that and chat about interesting things that go on in the day, and often during that time, I'll like bring up scenarios that happened and maybe ask for advice on how they would maybe more senior pharmacists would approach that scenario or what they think of it.”

The majority of participants specifically mentioned the workshop to be helpful (P2): “good doing the group session—brings up things that others would do differently, helpful if it presents in real life and you can apply those options”.

## Discussion

Hospital pharmacists participating in this study found the interactive education workshop on ethical decisions-relevant to their practice and beneficial in relation to developing their ethical reasoning skills. Our findings suggest that hospital pharmacists frequently rely on discussion with colleagues to think about how they resolve ethical issues. There was strong support among participants for more opportunities to discuss ethical challenges and ethical reasoning focusing on cases relevant to hospital pharmacy practice. Our findings align with earlier Australian research in this area by Chaar et al., although our cohort used peer support more [34]. This may be in part be a reflection of the location of our workshops, which were large well-staffed metropolitan hospitals.

Weighing risks and benefits of medication treatment options is part of routine practice for pharmacists, and pharmacists undertake considerable training dedicated to helping them do this safely and effectively. A comprehensive undergraduate curriculum supports pharmacists to weigh up the risks and benefits of medications in a patient centered way. This is followed by interns spending one year being supervised by a registered pharmacist who provides advice and feedback on how they deal with decisions around medications, including ethical scenarios. This all occurs prior to registration as a pharmacist.

Opportunities to engage in learning activities that simulate the complex decisions that pharmacists need to make in practice are difficult to simulate within pharmacy curricula. Practice experience and the need to take responsibility for professional decision-making within complex healthcare environments, sometimes in situations in which there are conflicting opinions or priorities, highlight the importance of bringing these kinds of workshop into the workplace. Haan et al. supports an interactive workshop design as it improves participants’ understanding of colleagues’ perspectives and participants feel more confident in responding to ethical challenges [19]. In addition, supervision from senior pharmacists may not assist early career pharmacists in dealing with ethical issues. More experienced pharmacists may have had as little training as less experienced pharmacists or may have developed a false confidence in their ethical approach through anchoring bias, or the illusory truth effect [35, 36].

Our research shows that codes of conduct from Australian pharmacy professional bodies and legislation are also utilised although somewhat less frequently. This has strengths and weaknesses. Professional body guidelines focus on legal aspects and may often guide pharmacists towards not dispensing medication and referring them to see their local general practitioner or in a hospital context the prescriber. Whilst this may be appropriate in many circumstances, in other circumstances this is merely shifting the ethical dilemma on to an even busier health practitioner.

Brief targeted ethical workshops that are contemporary and tailored to practice settings are valued by pharmacists and improve self-reported confidence in dealing with ethical issues. However, we acknowledge that our research does not identify whether these workshops are effective in actually increasing ethical behaviour. Introducing ethical workshops more generally within hospital pharmacists’ training and assessing the potential impact of these workshops on real life ethical decisions is warranted. This has also been called for by other authors in this area [37]. Workshops such as the one we describe have been identified as a platform that allows professionals to speak freely about issues without being judged, helps professionals build trust in one another, and improves confidence in ones’ practice, as experiences may be validated in these sessions [19]. Such workshops can also bring about understanding of colleagues and ones’ own perspectives on a moral issue alongside enhancing professionals’ sensitivity to moral issues, ultimately resulting in potential positive changes to patient care [19].

Integration with intern pharmacist training programs such as residencies, and undergraduate and postgraduate curricula is also required. Finally, our workshops were facilitated by pharmacists with formal bioethics training. We would advise these workshops be facilitated by people with formal ethics training in order for them to optimise learning in this area.

## Limitations

Limitations of our study is that this is a small study in metropolitan hospitals. Rural and remote hospitals are likely to have different ethical issues and their staff are likely to have different ethical training needs. Rural and remote pharmacists are unlikely to have access to more senior pharmacists with whom to discuss ethical issues. As this study involved self-assessment by participants, this may not reflect what the participants do when faced with ethical scenarios. Also, our study may be subject to self-selection bias. Participants who elected to attend the workshop are likely to have different needs to the general population of pharmacy staff. It is possible there may not have been the same value ascribed to these workshops if they were made mandatory for hospital pharmacy staff. Although this study used the same cases pre and post intervention to determine the value of the intervention, it may also have impacted the participants’ responses on the handling of the case.

## Conclusion

This interactive education workshop delivered to hospital pharmacists allowed us to consider a practical way to enhance pharmacists’ ethical reasoning skills in the workplace. The workshop allowed hospital pharmacists the opportunity to better understand how ethical decisions might be made and have shared understanding of values and goals. The findings of this study demonstrate that interactive education workshops like these could be used to enhance hospital pharmacists’ readiness and capabilities to recognise and respond to ethical issues and ultimately optimise patient healthcare.

### Supplementary Information

Supplementary Material 1.

Supplementary Material 2.

Supplementary Material 3.

## Data Availability

The datasets used and/or analysed during the current study are available from the corresponding author on reasonable request.

## Abbreviations

SHPA: Society of Hospital Pharmacists of Australia
PSA: Pharmaceutical Society of Australia
PBA: Pharmacy Board of Australia
STROBE: STrengthening the Reporting of OBservational studies in Epidemiology
COREQ: Consolidated Criteria for Reporting Qualitative Studies
MSHHS: Metro South Hospital and Health Service
GCHHS: Gold Coast Hospital and Health Service
PAH: Princess Alexandra Hospital
