# Supplementary material for: Evaluation of an interactive education workshop on hospital pharmacists’ ethical reasoning: an observational study
Source: BMC Med Ethics. 2024 Jul 23;25:81. doi: 10.1186/s12910-024-01082-4 (PMC11264360; doi:10.1186/s12910-024-01082-4)
Supplement: Supplementary file 1 — Supplementary Material 1. [file 12910_2024_1082_MOESM1_ESM.docx]

**Additional File 1**

***Framework for ethical decision-making (Adapted from Winch et al 2014)***

1.        Identify the problem and possible consequences of the problem

–         What facts are available?

–         What facts are still required?

–         What is the ethical problem? (Is there a conflict between two or more guiding principles?)

–         What harms are you concerned about? Who is affected?

2.        Identify relevant law and professional standards that apply to the case

–         What are your legal responsibilities?

–         What are your professional responsibilities?

–         How can you fulfil your legal and professional responsibilities in a way that brings about the best outcome in the situation?

3.        Identify the available options for resolving the problem and the reasons for or against each one

4.        Formulate a plan of action to resolve the problem

–         What do you need to put in place to improve the chances of a successful outcome?

–         How will you mitigate any risks you have identified?
