# Supplementary material for: Evaluation of an interactive education workshop on hospital pharmacists’ ethical reasoning: an observational study
Source: BMC Med Ethics. 2024 Jul 23;25:81. doi: 10.1186/s12910-024-01082-4 (PMC11264360; doi:10.1186/s12910-024-01082-4)
Supplement: Supplementary file 2 — Supplementary Material 2. [file 12910_2024_1082_MOESM2_ESM.docx]

**Additional File 2**

**Evaluation of ethical reasoning processes followed by hospital pharmacists**

**SURVEY**

**Overview**

The aim of this survey is to understand the reasoning and the processes that hospital pharmacists and interns apply to inform their decisions and actions when confronted with ethical dilemmas/issues. The information will help us identify gaps in ethical reasoning and develop training materials that address these gaps.

The survey is divided into Five sections, A, B, C, D and E. Section A collects demographics information, Section B - prioritising of resources, Section C - self-rated confidence with ethical reasoning, Section D contains vignettes with responses to practice scenarios, and Section E covers privacy and confidentiality scenarios.

The vignettes and scenarios you will encounter in this survey have been made intentionally ambiguous, dealing more with the ‘grey’ areas in practice rather than legal issues. Therefore, there are no right or wrong answers, just various perspectives and actions by pharmacists to solve these dilemmas/issues.

| Definitions of **ethics** and **ethical dilemmas**:  **Ethics:** moral principles that govern a person’s behaviour or the conducting of an activity; the moral correctness of specified conduct.^1^  **Ethical dilemmas:** a situation in which a difficult choice has to be made between two courses of action, either of which entails transgressing a moral principle.^2^ A “dilemma” becomes an “ethical dilemma” when the course of action involves uncertainty, conflicting values or may cause harm regardless of the action chosen. The most common type of ethical dilemma is the “uncertainty dilemma,” which refers to a problematic situation where “the right thing to do” is not clear, and where there are seemingly-equally valid reasons in support of two or more possible solutions to resolve the dilemma.^3^   1. Oxford Dictionary. Ethics. 2017; [https://en.oxforddictionaries.com/definition/ethics. Accessed 10/07/2017](https://en.oxforddictionaries.com/definition/ethics.%20Accessed%2010/07/2017). 2. Australian Defence Force. Definitions: Annex A to ADF Personal Conduct Review. 2011; <http://www.defence.gov.au/PathwayToChange/Docs/PersonalConductPersonnel/Review%20of%20Personal%20Conduct%20of%20ADF%20Personnel_appendices.pdf>. Accessed 10/07/2017. |
| --- |

**SECTION A: DEMOGRAPHIC INFORMATION**

1. **Mother’s initials and mother’s date and month of birthday: ………………………………….**
2. **Gender:** 🞎 Male 🞎 Female 🞎 Other 🞎 Prefer not to say
3. **Age group (years):** 🞎 21 – 30 🞎 31-40 🞎 41-50 🞎 51-60 🞎 >61
4. **Health service employment:** 🞎 GCHHS 🞎 MSHHS
5. **Position**: 🞎 Pharmacist 🞎 Intern

If pharmacist:

1. Years of practice experience as a hospital pharmacist:

🞎 0-2 🞎 > 2-5 🞎 > 5-10 🞎>10

1. Years worked as a pharmacist at current HHS:

🞎 0-2 🞎 > 2-5 🞎 > 5-10 🞎>10

1. **Current HP level: (Tick one only)**

- HP3 (intern)
- HP3
- HP4
- HP5
- > HP6

**SECTION B: RESOURCES**

1. **List UP TO five sources of support you are most likely to consult to inform your decision-making when presented with an ethical dilemma/issue in practice:**

| 1. **Indicate how often you use your knowledge around the following resources to inform your ethical decision making (either from memory or look up the resource):** | ***Daily*** | ***Weekly*** | ***Monthly*** | ***Less than monthly*** | ***Never*** |
| --- | --- | --- | --- | --- | --- |
| 1. Codes of Ethics and Conduct |  |  |  |  |  |
| 1. Standard for the Uniform Scheduling of Medicines Poisons (SUSMP, *Poisons Standard*) and relevant Commonwealth, state or territory drugs and poisons legislation |  |  |  |  |  |
| 1. Privacy Act (Cth) 1988 and privacy resources |  |  |  |  |  |
| 1. Legislation e.g. Medicines and Poisons Act (Qld) 2019 and Regulations 2021 |  |  |  |  |  |
| 1. Professional Competency Standards |  |  |  |  |  |
| 1. Professional Practice Standards |  |  |  |  |  |
| 1. Professional protocols or guidelines |  |  |  |  |  |
| 1. Pharmacy Board of Australia standards, codes and guidelines |  |  |  |  |  |
| 1. Workplace legal officer/team |  |  |  |  |  |
| 1. Workplace ethicist |  |  |  |  |  |
| 1. Co-workers in pharmacy department |  |  |  |  |  |
| 1. Co-workers in hospital outside of pharmacy department |  |  |  |  |  |
| 1. Discussion with colleagues via profession specific social media |  |  |  |  |  |
| 1. Staff at professional organisations (e.g. SHPA, PSA, Guild) |  |  |  |  |  |
| 1. Staff at the Pharmacy Board of Australia or AHPRA |  |  |  |  |  |
| 1. Professional indemnity insurer |  |  |  |  |  |

**SECTION C: ETHICAL REASONING**

| 1. **Please rate your level of agreement with the following statements:** | ***Strongly Disagree*** | ***Disagree*** | ***Neutral*** | ***Agree*** | ***Strongly Agree*** | ***Not sure*** |
| --- | --- | --- | --- | --- | --- | --- |
| 1. I am confident in identifying the regulatory frameworks applicable to pharmacy privacy requirements. |  |  |  |  |  |  |
| 1. I am confident in identifying the ethical issues applicable to pharmacy privacy requirements. |  |  |  |  |  |  |
| 1. I am confident in applying ethical reasoning to scenarios that involve pharmacy privacy dilemmas/issues. |  |  |  |  |  |  |
| 1. I follow a structured ethical decision-making process when confronted with ethical dilemmas/issues. |  |  |  |  |  |  |
| 1. I know where to find support when I have a clinical ethics question. |  |  |  |  |  |  |
| 1. I know where to find support when I have a non- clinical ethics question. |  |  |  |  |  |  |
| 1. I discuss ethically challenging scenarios in my hospital practice with my peers. |  |  |  |  |  |  |
| 1. Structured ethical decision-making skills impact positively on patient outcomes. |  |  |  |  |  |  |
| 1. Structured ethical decision-making skills impact positively on interactions with other health professionals. |  |  |  |  |  |  |
| 1. I am regularly presented with clinical ethics scenarios in my practice as a hospital pharmacist or intern. |  |  |  |  |  |  |
| 1. I am regularly presented with non-clinical ethics scenarios in my practice as a hospital pharmacist or intern. |  |  |  |  |  |  |
| 1. I would participate in quarterly ethics cafes (interactive small group discussions) if these were made available. |  |  |  |  |  |  |

**SECTION D: VIGNETTES**

**Palliative care patient and high dose of opioid**

You are undertaking an inpatient medicine review of a patient with end-stage metastatic cancer and notice an irregular pattern of opioid administration, although it has been prescribed as regular and not prn. The patient’s notes states that she suffers from severe abdominal pain with opioids, the only medicines that are effective at this stage to provide some pain relief. The patient is regarded as competent to make her own decision.

When you discuss this with the nurse, she informs you that the patient’s husband does not want her to take any sedating pain medicines as he does not want her to be ‘drowsy and confused’ and adds that his nephew was previously addicted to prescription pain killers. He is preventing her from receiving pain medication and the patient therefore refuses to take her medicines during the day when her husband is there at her bedside. However, when he is not there at night, she requests the opioid and appears to be much more comfortable.

When you talk to the patient during your inpatient unit review one morning, (the husband is not there, he has left to get a coffee), the patient tells you that her pain is intolerable, but she wants to ‘please’ her husband. You have another discussion with the nurse who tells you she has been ‘sneaking in’ when the husband is not there to administer some medicines as the patient is a competent decision maker.

| 1. **Please indicate your level of agreement with the following hypothetical options you could undertake.**   **You**: | ***Strongly Disagree*** | ***Disagree*** | ***Neutral*** | ***Agree*** | ***Strongly***  ***Agree*** | ***Not sure*** |
| --- | --- | --- | --- | --- | --- | --- |
| 1. … agree with the nurse and would suggest she keeps doing the same thing with no need to inform the husband |  |  |  |  |  |  |
| 1. … agree with the nurse and would suggest she keeps doing the same thing but insist the nurse informs the husband about this |  |  |  |  |  |  |
| 1. … disagree with the nurse’s behaviour but do not interfere with the process as it is not your role |  |  |  |  |  |  |
| 1. … you inform the husband of the situation as he has legal say as the next of kin, do not report the incident |  |  |  |  |  |  |
| 1. … you inform the husband of the situation as he has legal say as the next of kin, report the incident |  |  |  |  |  |  |
| 1. … you do not discuss the situation with the patient or husband and Riskman or report an incident |  |  |  |  |  |  |
| 1. … you discuss your concerns with the patient but not the husband |  |  |  |  |  |  |
| 1. … you discuss your concerns with the patient and her husband |  |  |  |  |  |  |
| 1. … you discuss the situation with a senior medical officer |  |  |  |  |  |  |
| 1. … you discuss the situation with another pharmacist |  |  |  |  |  |  |

**Please provide comments on the above scenario:**

________________________________________________________________________________

________________________________________________________________________________

________________________________________________________________________________

**Disagreeing with doctor’s decision**

You have been handed a discharge prescription for antibiotic ZX for an elderly gentleman. On reading his notes you see that it has not yet been started in hospital, but the doctors are happy for him to go home and start taking the antibiotic for his skin infection.

You find out that he has had a medicine from the same class before, Drug ZY, and states it caused bad nausea and vomiting, resulting in him not completing the antibiotic course. Drug ZX and Drug ZY have the same mechanism of action and similar adverse effect profiles.

You contact the discharge doctor, and she informs you that she was aware of the elderly gentleman’s previous experience with Drug ZY, but she considered other possibilities and is content with her choice of medication. The doctor informs you she has met the patient on previous admissions, and in her opinion his adverse reaction is “not real, it’s all in his mind”.

| 1. **Please rate your level of agreement with the following hypothetical options you could undertake.**   **You continue with discharge preparation**: | ***Strongly Disagree*** | ***Disagree*** | ***Neutral*** | ***Agree*** | ***Strongly Agree*** | ***Not sure*** |
| --- | --- | --- | --- | --- | --- | --- |
| 1. … without saying anything to the patient because you do not want to discredit the doctor. |  |  |  |  |  |  |
| 1. … without saying anything to the patient because you accept the doctor’s explanation of the adverse reaction being all in the patient’s mind. |  |  |  |  |  |  |
| 1. … without saying anything to the patient as his previous adverse reaction was not serious. |  |  |  |  |  |  |
| 1. … without saying anything to the patient as it is more important for the patient to be compliant with his medication and telling him of the side effects may result in him being non-compliant. |  |  |  |  |  |  |
| 1. … and inform the patient that both medications are similar and therefore he may experience the same response as previously. |  |  |  |  |  |  |
| 1. … and you inform the patient that the medications are not similar and therefore it is unlikely for him to experience the same response as previously. |  |  |  |  |  |  |
| 1. … but discuss the situation with another pharmacist |  |  |  |  |  |  |
| 1. … but discuss the situation with another medical officer |  |  |  |  |  |  |
| 1. … but document your concerns and actions |  |  |  |  |  |  |

**Please provide comments on the above scenario:**

________________________________________________________________________________

________________________________________________________________________________

________________________________________________________________________________

**SECTION E: Privacy and confidentiality scenarios**

| 1. **In your regular practice, how often are you exposed to the following scenarios?** | ***Daily*** | ***Weekly*** | ***Fortnightly*** | ***Monthly*** | ***Less than monthly*** | ***Never*** |
| --- | --- | --- | --- | --- | --- | --- |
| 1. The pharmacy receives a fax from a health professional intended for another receiver. The fax contains patient identifiers/ information. |  |  |  |  |  |  |
| 1. A staff member relays medicines information to a family member and then realises that he/she may not be entitled to that information. |  |  |  |  |  |  |
| 1. A staff member shows one member of a family another family member’s discharge medication record without obtaining consent from them. |  |  |  |  |  |  |
| 1. A patient is counselled on their medicines in front of a family member without obtaining their consent first. |  |  |  |  |  |  |
| 1. A patient requests you or another staff member to withhold recording some of their medicine in ieMR. |  |  |  |  |  |  |
| 1. A staff member discusses confidential de-identified information about a consumer outside of the pharmacy at a non-professional setting. |  |  |  |  |  |  |
| 1. A staff member discloses confidential identifiable information about a consumer(s) outside of the pharmacy. |  |  |  |  |  |  |
| 1. A staff member discloses real practice scenarios on a social media platform such as Facebook. |  |  |  |  |  |  |
| 1. A staff member sorts through prescriptions on a front/dispensary counter in view of other consumers. |  |  |  |  |  |  |
| 1. Identifiable patient and/or consumer information disposed of in unsecured rubbish (e.g. note) |  |  |  |  |  |  |
| 1. Empty, used dose administration aid (DAA) packs with identifiable header cards and medicine details are disposed of in unsecured rubbish. |  |  |  |  |  |  |
| 1. Medicines awaiting collection have dispensing labels visible to other consumers. |  |  |  |  |  |  |
| 1. A staff member on an in-patient unit leaves the computer screen on with identifiable patient information visible. |  |  |  |  |  |  |
| 1. Medicines handed out are visible to other patients in the in-patient unit. |  |  |  |  |  |  |
