# Supplementary material for: Evaluation of an interactive education workshop on hospital pharmacists’ ethical reasoning: an observational study
Source: BMC Med Ethics. 2024 Jul 23;25:81. doi: 10.1186/s12910-024-01082-4 (PMC11264360; doi:10.1186/s12910-024-01082-4)
Supplement: Supplementary file 3 — Supplementary Material 3. [file 12910_2024_1082_MOESM3_ESM.docx]

**Additional File 3**

**Evaluation of ethical reasoning processes followed by hospital pharmacists**

**SEMI-STRUCTURED INTERVIEW**

**Opening Remarks:**

*Thank you for your time.*

*I would like to start off with a brief introduction.*

*The aim of this interview is to understand the reasoning and the processes that hospital pharmacists and interns apply to inform their decisions and actions when confronted with ethical dilemmas. The information will help us identify gaps in ethical reasoning and develop materials that address these gaps.*

*The scenarios you will encounter in this interview raise difficult issues, dealing more with the ‘grey’ areas in practice rather than legal issues*

*We really value your perspective. Anything you tell us will be treated with the strictest of confidence and your identity will be protected.*

*If you have any questions regarding this research, please do not hesitate to contact any of the research team members*.

| Definitions of **ethics** and **ethical dilemmas**:  **Ethics:** moral principles that govern a person’s behaviour or the conducting of an activity; the moral correctness of specified conduct.^1^  **Ethical dilemmas:** a situation in which a difficult choice has to be made between two courses of action, either of which entails transgressing a moral principle.^2^ A “dilemma” becomes an “ethical dilemma” when the course of action involves uncertainty, conflicting values or may cause harm regardless of the action chosen. The most common type of ethical dilemma is the “uncertainty dilemma,” which refers to a problematic situation where “the right thing to do” is not clear, or it is clear what the “right thing” to do is, but it is hard to do; and where there are seemingly-equally valid reasons in support of two or more possible solutions to resolve the dilemma.   1. Oxford Dictionary. Ethics. 2017; [https://en.oxforddictionaries.com/definition/ethics. Accessed 10/07/2017](https://en.oxforddictionaries.com/definition/ethics.%20Accessed%2010/07/2017). 2. Australian Defence Force. Definitions: Annex A to ADF Personal Conduct Review. 2011; <http://www.defence.gov.au/PathwayToChange/Docs/PersonalConductPersonnel/Review%20of%20Personal%20Conduct%20of%20ADF%20Personnel_appendices.pdf>. Accessed 10/07/2017. |
| --- |

***The interview should take about 30 minutes.***

**DEMOGRAPHIC INFORMATION**

1. **Gender:** 🞎 Male 🞎 Female 🞎 Other 🞎 Prefer not to say
2. **Age group (years):** 🞎 21 – 30 🞎 31-40 🞎 41-50 🞎 51-60 🞎 >61
3. **Position**: 🞎 Intern 🞎 Pharmacist

If pharmacist: Total years of practice experience as a hospital pharmacist: __________ years

How long worked as a pharmacist at GCHHS/MSHHS:______________ years

1. **Current HP level: (Tick one only)**

- HP3 (intern)
- HP3
- HP4
- HP5
- > HP6

1. Did you attend the recent education session on pharmacist’s ethical reasoning skills delivered at GCH or MSHHS?

If yes, can you please give me some feedback: process, case, group size etc.

1. Can you please provide a brief outline of your current position and what this involves?

How long have you worked in this position?

What are the current areas you work in? If there are multiple areas please specify e.g. clinics, inpatient units.

What proportion of time is spent in these areas?

Have you supervised pharmacy students? How often?

1. What do you understand to be the difference between ethical issues and ethical dilemmas?

How often do you encounter ethical issues?

How important to your day to day practice is being able to identify and resolve ethical issues?

1. Where and how did you develop your ethical reasoning skills and processes?

What were the most influential experiences?

1. How often do you think about your ethical practice?

Is ethical practice something that is a constant factor in your mind, an underlying characteristic of how you practise, or something you rarely think about?

1. Could you estimate how frequently ethical issues arise when practising as a hospital pharmacist?
2. Could you estimate how frequently you are confronted with ethical dilemmas when practising as a hospital pharmacist?

**SCENARIO 1**

A 70 year old homeless male patient is ready to be discharged from hospital. He has been admitted after having a fall and sustaining a fractured neck of femur. His discharge prescriptions are to be dispensed at the hospital pharmacy. His current discharge prescriptions are Oxycontin SR (oxycodone SR) 40mg, 1 twice a day (quantity 28, no repeats) and Oxycodone 5mg, 1-2 every 4 hours when required (quantity 10).

Using Q Script, you notice that, prior to coming into hospital, he has obtained regular supplies of oxycodone prescribed by different general practitioners and supplied by different community pharmacies. His transport will be arriving in 20 minutes.

*Discuss some ethical considerations surrounding this scenario?*

**SCENARIO 2**

**Sotrovimab (or latest evidence that is available)** is a monoclonal antibody that is used to treat people at risk of serious covid related illness. It has been shown to reduce hospitalization or death by 79% in adults with mild to moderate COVID-19, at risk of developing serious illness.

It is a single dose, delivered via intravenous infusion. Patients will receive treatment free of charge through the public health system.

The Australian Government has secured at least 7700 doses of Sotrovimab.

At the time it was first being used, under NSW guidelines, patients were **ONLY** be eligible for treatment if they were not fully vaccinated.

*Discuss some ethical considerations surrounding the use of Sotrovimab in this setting.*

*(Prompts if information not forthcoming)*

- *Cost to health service*
- *Impact on treating health professionals*

References:

[Model of care for the use of sotrovimab in adults in NSW - Communities of practice](https://www.health.nsw.gov.au/Infectious/covid-19/communities-of-practice/Pages/model-care-adults-sotrovimab.aspx)

[TGA approves new COVID-19 treatment for use in Australia | Health Portfolio Ministers](https://www.health.gov.au/ministers/the-hon-greg-hunt-mp/media/tga-approves-new-covid-19-treatment-for-use-in-australia)

**SCENARIO 3**

You are a pharmacist working on a respiratory ward. You receive a CD order request for phenobarbitone injection. The patient is prescribed a very high dose of 1200mg daily via subcutaneous intravenous infusion to start immediately. You ask the resident what this is for, and the resident says the patient is palliative. You find a guideline from NSW health for ‘Subcutaneous phenobarbital for refractory terminal agitation and uncontrolled seizures (including status epilepticus) in the imminently dying patient’ that recommends this dose. You ask the resident if the patient is agitated or having seizures. The resident says, “not at the moment, but they could become agitated or have seizures in the future if this is not started.”

*What is your approach to this situation?*

*Prompts:*

- *Get initial response; if the pharmacist is not clear that this dose is likely to lead to death; discuss likely outcome and see if participant changes their response*
- *What are your thoughts around your professional responsibility in supply?*
- *What are your thoughts around discussing this with the resident/treating team?*
- *What are your thoughts around discussing this with the family?*
